# Correcting Duporcq's theorem^☆^

**DOI:** 10.1016/j.mechmachtheory.2013.11.012

**Published:** 2014-03

**Authors:** Georg Nawratil

**Affiliations:** Institute of Discrete Mathematics and Geometry, Vienna University of Technology, Wiedner Hauptstrasse 8–10/104, Vienna A-1040, Austria

**Keywords:** Borel Bricard problem, Architectural singularity, Stewart–Gough platform, Self-motion

## Abstract

In 1898, Ernest Duporcq stated a famous theorem about rigid-body motions with spherical trajectories, without giving a rigorous proof. Today, this theorem is again of interest, as it is strongly connected with the topic of self-motions of planar Stewart–Gough platforms. We discuss Duporcq's theorem from this point of view and demonstrate that it is not correct. Moreover, we also present a revised version of this theorem.

## Highlights

• We study the connection between Duporcq’s theorem and architecture singularity.
• Based on this point of view, we demonstrate that Duporcq’s theorem is not correct.
• Moreover, we present a revised version of Duporcq’s theorem.

## 1 Introduction

On May 16th, 1898 the following theorem of Ernest Duporcq was published in [1], which was written in French:

Theorem 1 Si un plan P se déplace dans l'espace de sorte que cinq de ses points restent sur des sphéres dont les centres appartiennent à un plan fixe P′, il existera dans le plan P un sixième point jouissant de la même propriété.

A correct – close to a word-by-word – translation of this theorem into English, was given by Emch [2]:

Translation 1 If five points of a plane P move on five fixed spheres whose centers lie on a fixed plane P′, then there exist on P a sixth point which also describes such a sphere.

A similar translation was also used by Forder in Example 40 of [3]. A further, more loose English translation of this theorem was given by Husty [4]:

Translation 2 Given five points in a plane of the moving system and five points in a plane of the fixed system, then there exists an additional unique pair of points which will remain at a fixed distance in the motion induced by the five other pairs.

This translation is not totally correct as the sixth pair has not to be unique (cf. Translation 1). A corrected version of Husty's translation was used by Karger [5]:

Translation 3 Let us have five coplanar points in the moving space and five coplanar points in the fixed space. Then there exists another pair of points which remain at fixed distance during the motion determined by five others.

This corrected version is still a bit inaccurate, as it does not contain the fact, that the sixth point pair has to be located in the planes spanned by the given five point pairs. Therefore, we prefer Translation 1 of Emch for the remainder of this article.

Duporcq's theorem gained importance six years after his publication, as in 1904, the French Academy of science posed the following problem for the *Prix Vaillant* (cf. Husty [4]): *Determine and study all displacements of a rigid body in which distinct points of the body move on a spherical path*. Nevertheless, the papers of Borel [6] and Bricard [7]^2^ only presented partial solutions, they were awarded prizes. This still unsolved problem, which is also known as the Borel Bricard problem, attracted new attention within the last years in the context of self-motions of parallel manipulators of Stewart–Gough (SG) type (cf. [4], [5], [8]).

This important theoretical issue in the singularity analysis of SG platforms is also our motivation for focusing on Duporcq's theorem within this article. In Section 2, we give an English translation (in extracts) of Duporcq's arguments given in [1], which incite him to formulate the theorem under consideration. Moreover, we list three gaps within Duporcq's argumentation, which give rise to the need to take a closer look on this theorem. Based on this review, the relation between Duporcq's theorem and architecturally singular Stewart–Gough manipulators, with a planar platform and planar base, is clarified in Section 3. Section 4 is devoted to the “*projective closure*” of Duporcq's theorem. In Section 5, we prove a corrected version of Duporcq's theorem. Finally, in Section 6, we demonstrate on the basis of a concrete example, which is also a counter example to Duporcq's theorem, that the given corrected version is the most general one.

## 2 Duporcq's argumentation

**Fig. 1:**
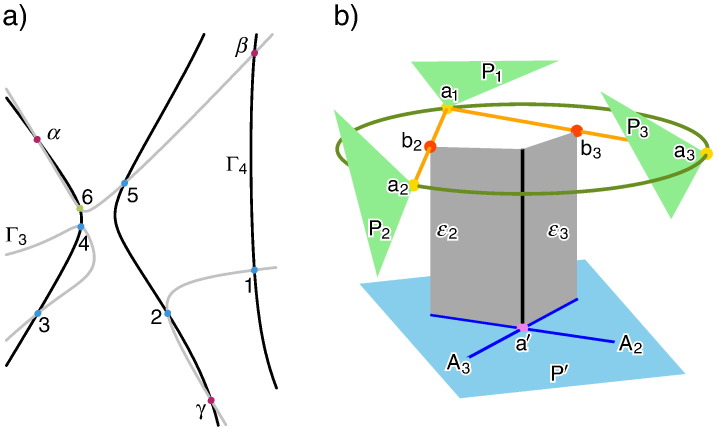
a) Sketch of the cubics *Γ*_3_ and *Γ*_4_ and their intersection points 1, …, 6, *α*, *β*, *γ*. b) It is well known (cf. page 166 of [10]), that the bisecting planes *ε*_*i*_ of corresponding points a_1_ ∈ P_1_ and a_*i*_ ∈ P_*i*_ are related with the midpoints b_*i*_ of the segment a_1_a_*i*_ by a null-polarity (for *i* = 2, 3). Therefore, the mapping from a ∈ P to the intersection line A_i_ of *ε*_*i*_ an P′ is a correlation. The intersection point of A_2_ and A_3_ is equivalent with the intersection point of the axis of the circumcircle of a_1_, a_2_, a_3_ and P′.

In the following we give a – close to a word-by-word – translation of those parts of Duporcq's paper [1] (see also [9]^3^), which are of interest for us. The first three paragraphs of [1] read as follows (cf. Fig. 1a):

Assume (a, A_1_) and (a, A_2_) are two correlations, which assign to each point a of the plane P the corresponding lines A_1_ and A_2_ of the plane P′, respectively. The intersection point of A_1_ and A_2_ is denoted by a′. It can easily be seen, that in the general case, the pointwise transformation (a, a′) is defined by seven pairs of corresponding points. Moreover, it is known that the lines A_1_ and A_2_ coincide for three poses *α*, *β* and *γ* of the point a. The transformation (a, a′) is a rational quadratic transformation.^4^
Moreover, we consider an analog transformation (a, a″), which results from the two correlations (a, A_3_) and (a, A_4_). It can be verified without difficulties, that the lines A_1_, A_2_ and A_3_ of the plane P′ are copunctal, if the point a belongs to a cubic Γ_4_, which contains the points *α*, *β* and *γ*. The analogously defined cubic Γ_3_ (set of points a, where the corresponding lines A_1_, A_2_ and A_4_ are copunctal) intersects the cubic Γ_4_ in six points 1,…,6 different from *α*, *β* and *γ*, where the correspondents 1′, …, 6′ and 1″, …, 6″, with respect to the transformations (a, a′) and (a, a″), respectively, coincide pairwise (i.e. 1′ = 1″, …, 6′ = 6″).
Hence, we consider all rational quadratic transformations (a, a″), for which the points 1′, …, 5′ and 1″, …, 5″ coincide pairwise: All cubics, which pass through the five points 1,…,5 as well as through the points *α*, *β* and *γ*, have a ninth point 6 in common. Due to the previous result, the point 6′ has to coincide with 6″.

Now, we skip a few paragraphs in Duporcq's paper [1], and proceed with the part where he noted the following interesting application of the above given result. Duporcq's explanations are illustrated and commented on in Fig. 1b:

P_1_, P_2_ and P_3_ are three poses of a plane P in the Euclidean 3-space and P′ is a fixed plane. Moreover, we denote the corresponding points of a point a of P by a_1_, a_2_ and a_3_, respectively. Now, we get a rational quadratic transformation, if we assign to each point a, the intersection point of the plane P′ and the axis of the circumcircle of the three points a_1_, a_2_ and a_3_.

In Duporcq's mind, this consideration, together with the above given result, proves Theorem 1. In the part of Duporcq's paper [1], which was omitted in the above given translation, only one special case was discussed by the author. Duporcq showed that for this special case the statement of Theorem 1 could even be strengthened as follows:

Theorem 2 If five points of the plane P and the corresponding coplanar centers are within a projectivity κ, then each point a of the conic c defined by the five points have a fixed distance to its corresponding point aκ of the conic cκ of P′.

For Duporcq, this result also confirmed the validity of Theorem 1, as Theorem 2 was already known to Chasles [11] within a static context and to Bricard (cf. footnote 1 of [1] and footnote 2 on page 3 of [7]).

### 2.1 Argumentative gaps in the proof of Duporcq

In the following, we list three gaps within Duporcq's argumentation:

Gap 1 Is it possible, that the point 6 or the point 6′, respectively, does not exist from the Euclidean point of view, as it is a point at infinity (= ideal point)?
Gap 2 Is it possible, that the sixth point pair (6, 6′) does not exist, as it coincides with one of the given five point pairs (i, i′) for i ∈ {1,…,5}?
Gap 3 What happens if at least four points of {1,…,6} or {1′, …,6′} are collinear? For these cases the argumentation based on cubics fails completely, as two cubics can only intersect in three points along a line.

## 3 Duporcq's theorem in the context of planar SG manipulators

**Fig. 2:**
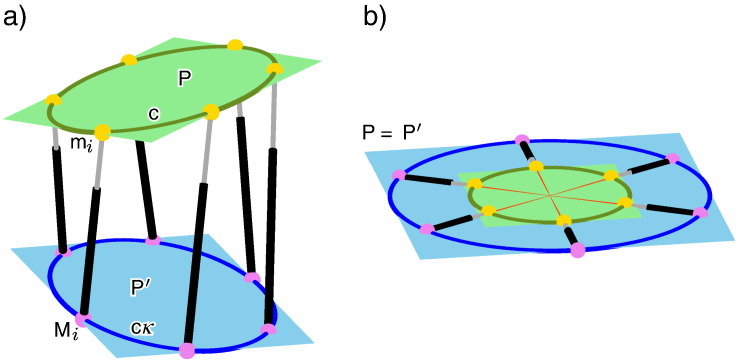
a) A planar architecturally singular SG platform is given, where the platform anchor points m_*i*_ and the base anchor points M_*i*_ are related by a non-singular projectivity *κ*; i.e. m_*i*_*κ* = M_*i*_ for *i* = 1, …, 6 (cf. Theorem 2). b) It is well known (e.g. Section 3.1 of [12]), that planar architecturally singular SG platforms are redundant. Therefore they possess a self-motion in each pose. It can easily be seen by the above given example, that this only holds over ℂ: In the illustrated pose the platform and the base coincide as well as the centers of the two circles c and c*κ* (*κ* is a similarity).

A Stewart–Gough (SG) platform is a parallel manipulator consisting of a moving platform, which is connected via six spherical–prismatic–spherical legs with the base, where the spherical joints are passive and the prismatic joints are active. Therefore, the geometry of a SG platform is given by the six base anchor points M_i_ and by the six platform anchor points m_i_ for *i* = 1,…,6. A SG platform m_1_, …, M_6_ is called planar if m_1_, …, m_6_ as well as M_1_, …, M_6_ are coplanar (cf. Fig. 2a).

If the geometry of the SG platform and the six leg lengths are given, the manipulator is in general rigid in one of its 40 possible solutions of the direct kinematic problem. But, under particular conditions the manipulator can perform an *n*-parametric motion (*n* > 0), which is called self-motion and corresponds to an *n*-dimensional solution of the forward kinematics. Clearly, in each pose of a self-motion the SG platform is singular; i.e. the carrier lines of the prismatic legs belong to a linear line complex (cf. [13]). Finally, it should be noted, that SG manipulators, which are singular in every possible configuration, are so-called architecturally singular SG platforms (see Fig. 2).

Now, we look back on Duporcq's arguments given in Section 2: The planes P_1_, P_2_ and P_3_ are three poses of the planar platform and P′ is the planar base. Moreover, a′ is the base anchor point of the corresponding platform anchor point a. If the construction is done in the way described by Duporcq, then also the leg length (sphere radius) is determined for each of the five legs. But the determination of the sixth point pair only depends on the given five point pairs and not on the leg lengths (sphere radii). Therefore, the resulting SG manipulator has to have a self-motion in each pose of the platform, which already yields that this manipulator has to be an architecturally singular one. Therefore, Duporcq's theorem can be translated into the language of parallel manipulators of SG platforms as follows:

Translation 4 If five pairs of anchor points of a planar manipulator are given, then there exists a sixth point pair in a way that the resulting planar architecturally singular SG platform has the same solution for the direct kinematics as the given 5-legged manipulator.

**Fig. 3:**
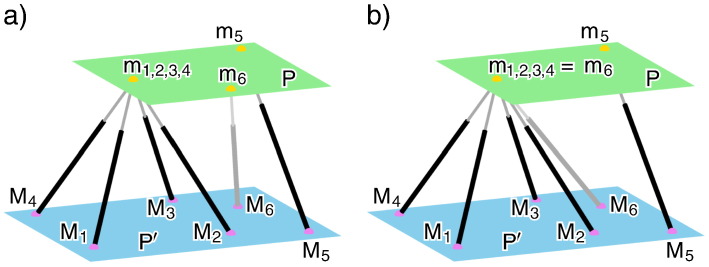
Given is a 5-legged manipulator with m_1,2,3,4_ := m_1_ = m_2_ = m_3_ = m_4_. This manipulator is degenerated (cf. [14]) and possess a two-parametric spherical self-motion with center m_1,2,3,4_. By adding any leg with anchor points m_6_ ∈ P and M_6_ ∈ P′, we always obtain a planar architecturally singular SG platform: a) In the general case, the sixth leg restricts the self-motion to a one-dimensional one. b) If we choose m_6_ = m_1,2,3,4_, the resulting SG platform has the same solution for the direct kinematics as the given 5-legged one.

Remark 1 Assume that the given 5-legged manipulator is not degenerated (cf. [14]), i.e. the carrier lines of the five legs do not belong to a linear congruence of lines (cf. page 173 ff. of [10]) in each configuration. Then it is well known (e.g. Section 3.1 of [12]), that the resulting architecturally singular SG platform of Translation 4 is redundant, which already implies that it has the same solution for the direct kinematics as the given 5-legged manipulator. Therefore, this apposition only affects the case of degenerated 5-legged manipulators. Its consequences are explained with the help of an example illustrated in Fig. 3. ◊

The author is very sure that Duporcq wanted his theorem to be understood in the way of Translation 4, but if we purely translate Duporcq's theorem, without taking his argumentation into account, we end up with the following more general version:

Translation 5 If five pairs of anchor points as well as the five leg lengths of a planar manipulator are given, then there exists a sixth point pair with a corresponding leg length in a way that the resulting planar SG platform has the same solution for the direct kinematics as the given 5-legged manipulator.

Remark 2 In Translation 5, the sixth point pair can change with respect to different values for the lengths of the first five legs. In Translation 4, the sixth point pair is independent with respect to these variables. ◊

In this article, we want to show that Theorem 1 is not valid, which is now equivalent with the task of finding a counter example to the statement given in Translation 5. Clearly, this counter example also has to falsify Translation 4. For the determination of this example, we go the other way round. In a first step, we determine the set of five point pairs for which Translation 4 does not hold. This is done within the proof of the corrected version of Duporcq's theorem (cf. Theorem 5). Based on this result, we construct a concrete counter example to Translation 5 in Section 6.

In view of the first step, we proceed with a detailed review on architecturally singular SG manipulators.

### 3.1 Review on architecturally singular SG manipulators

For a serious review on this topic, one has to distinguish between two approaches for the determination of the set A of architecturally singular parallel manipulators; the work done by Karger and Husty on the one hand, and the approach of Röschel and Mick on the other hand.

#### 3.1.1 Results of Karger and Husty

These two authors wrote a series of papers (e.g. Karger [14], [15] and Husty and Karger [16], [17]) on this topic, which finally end up in the two main contributions [18], [19].

Karger presented in Theorem 1 of [18] the four sufficient and necessary conditions for architecturally singular planar SG platforms with no four anchor points aligned. Beside this algebraic characterization, also a projective geometrical one was given. Moreover, Karger proved in Theorem 1, Theorem 2 of [19], that architecturally singular non-planar SG platforms have four collinear anchor points. Finally, in Theorem 3 of [19], all types of architecturally singular manipulators with four collinear anchor points were listed. This list also contains the so-called degenerated planar cases, which were not treated in [16]. Moreover, in [16], [19] a geometric interpretation of the listed designs (with exception of the degenerated planar cases; cf. [20]^5^) was given.

Considered in retrospect, one can say that this approach is based on the subdivision of the set A into two classes with respect to the criterion of possessing four collinear anchor points or not.

#### 3.1.2 Results of Röschel and Mick

Based on the results of Karger [15], another attempt for the determination of A was done by Röschel and Mick [21], [22]. They divided this set into planar and non-planar manipulators, but they were only able to give an analytic (cf. Remark 1 of [22]) and a geometric characterization (cf. Theorem 4.2 of [22]) for the planar case. The latter reads as follows:

Theorem 3 Planar SG platforms are architecturally singular iff the point pairs (M*_i_*,m*_i_*) for *i* = 1,…,6, are four-fold conjugate pairs of points with respect to a 3-dimensional linear manifold of correlations or one of the two sets {M*_i_*} and {m*_i_*} of anchor points is aligned.

Note, that a similar characterization for the general case (planar and non-planar) was given by the author in [23] by subdividing A with respect to the criterion, whether the legs belong in every possible configuration to a singular linear line complex^6^ or not.

Remark 3 For the sake of completeness, it should also be mentioned that further articles about architecturally singular parallel manipulators were published by several researchers. In this context, we only want to mention Wohlhart's approach [24]. He determined architecturally singular planar SG manipulators by adjusting the anchor points of the sixth leg, with respect to the criterion, that the resulting manipulator has to be singular to the fourth degree in an arbitrarily chosen pose. ◊

Finally, it should be pointed out, that Duporcq's argumentation (cf. Section 2) fits with the result given in Theorem 3. The six point pairs (1,1′), …, (6,6′) are conjugated with respect to the correlations (a,A_1_), …, (a,A_4_) and therefore these six pairs of points also form an architecturally singular manipulator, if the matrices of the four correlations (a,A_1_), …, (a,A_4_) are linearly independent.

## 4 Projective closure of Duporcq's theorem

It is well known (e.g. Remark 4, Theorem 4.2 of [22]), that architecturally singular manipulators remain architecturally singular, if we apply projectivities *μ* and *μ*′ to the platform and the base, respectively. Clearly, this property can be used to falsify Translation 4 as follows.

We start with an SG platform m_1_, …, M_6_ of the set *ℳ* of planar architecturally singular manipulators with no four points collinear, where the platform anchor points and the base anchor points are not related by a projectivity (cf. Theorem 2). Then, we apply a regular projectivity *μ* to the platform, which maps the anchor point m_6_ to infinity and keeps the points m_1_*μ*, …, m_5_*μ* finite. Moreover, we choose *μ*′ as any regular projectivity, such that the six points M_1_*μ*′, …, M_6_*μ*′ are finite; e.g. *μ*′ is the identity.

If we now consider the points M_1_*μ*′, …, M_5_*μ*′ in the base and m_1_*μ*, …, m_5_*μ* in the platform and ask for the sixth point pair in the sense of Translation 4, we would end up with the unique solution (m_6_*μ*, M_6_*μ*′). The uniqueness of the result is due to the following theorem, which was proven by the author in [8]:

Theorem 4 To any planar SG platform with exception of the set *ℳ*, at least a one-parametric set of legs can be attached without changing the forward kinematics.

The constructed example belongs to Gap 1 (cf. Section 2.1) as m_6_*μ* is an ideal point.^7^ Therefore, this is a counter example to Translation 4. Karger gave a concrete example for this circumstance in [5], where he also presented a modified version of Duporcq's theorem, which is based on results obtained in [18] and on the exclusion of the case that the anchor points of the sixth point pair are ideal points. For details we refer to [5], [25]. In contrast to Karger, we do not want to restrict Duporcq's theorem, but we want to extend it with respect to the projective closure of the Euclidean 3-space. In order to close Gap 1, we have to clarify the question, how the condition, that a point m is located on a sphere with center M (=*sphere condition*), transforms, if the point m or the center M, or both, are ideal points. This is done in the next subsection.

### 4.1 The Darboux, Mannheim and angle condition

**Fig. 4:**
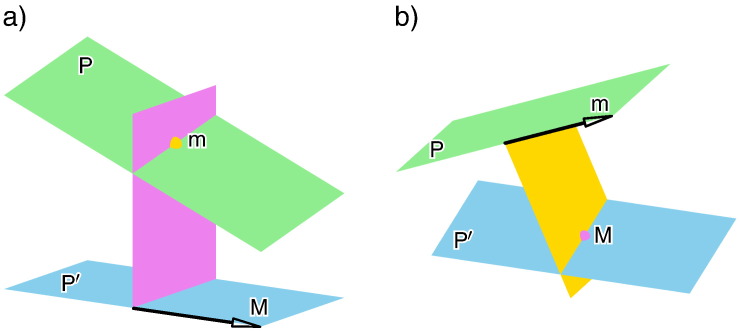
a) Sketch of the Darboux condition. b) Sketch of the Mannheim condition.

Classically, a sphere with center M trough m is defined as the set of all points, which have the same distance from M as m. By considering a limiting process, it can be verified without difficulties, that the sphere condition degenerates in the so-called *Darboux condition* (cf. [12]), if the center M of the sphere goes to infinity and m remains finite. This means that the point m is located in a fixed plane orthogonal to the direction of the ideal point M (see Fig. 4a).

By changing the role of the platform and the base, it can easily be seen, that the sphere condition degenerates in the so-called *Mannheim condition* (cf. [12]), if the point m goes to infinity and M remains finite. This means that a plane of the moving system, orthogonal to the direction of the ideal point m, slides through the point M (see Fig. 4b). Note that the Mannheim motion is the inverse of the Darboux motion (cf. [26]).

By tackling the problem by means of a limiting process, we get argumentative difficulties for the case that both points move to infinity simultaneously. These troubles can be solved by using the following definition for the sphere: The set of points, which are obtained by reflecting m on all finite planes through M. The advantage of this point of view is, that the definition does not depend on the sphere radius.

The elegance of this approach can be seen by putting the rule to the test at the basis of the Darboux condition: If M is an ideal point, we have to reflect the finite point m on all finite planes through M. The resulting set of obtained points is trivially the plane through m orthogonal to M. Therefore, we get exactly the Darboux condition. By regarding the inverse problem, we clearly end up with the Mannheim condition.

**Fig. 5:**
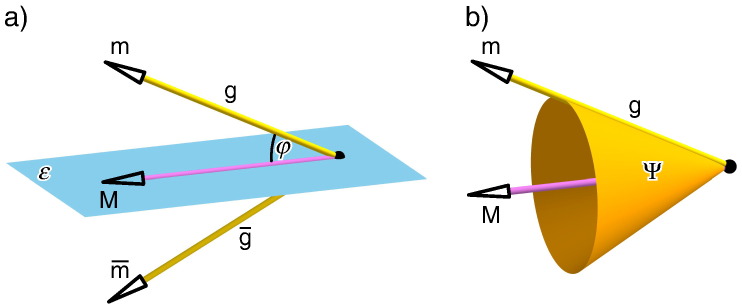
a) The reflection of m, with respect to an finite plane *ε* through M, can easily be done by considering m as the ideal point of a line g. Then the reflected point $\bar{m}$ is the ideal point of the reflected line $\bar{g}$ with respect to *ε*. b) The obtained set of points $\bar{m}$ are the ideal points of a cone of revolution *Ψ* with half apex angle *φ* and where M is located on the axis of revolution.

Now, we use this approach for the clarification of the open case that both points M and m are ideal points. Again we reflect m on all finite planes through M (cf. Fig. 5a). The resulting set of ideal points, trivially has the property that each point enclose the same angle *φ* with the ideal point M (cf. Fig. 5b). Therefore, we call this condition *angle condition*.

Remark 4 This result is also confirmed by the work [27] of the author, where he showed that during each self-motion of a non-architecturally singular SG platform, where the planar platform and the planar base are related by a projectivity, the angle enclosed by corresponding ideal points (with respect to the projectivity) remains constant. This is also the reason why SG platforms, where the planar platform and the planar base are related by an affinity, can only have translational self-motions (cf. [28]).

Moreover the reviewer of [27] has drawn the author's attention to Chapter II (item 6 and item 7) of Bricard [7], where an algebraical proof for the Darboux condition and the angle condition can be found. ◊

## 5 Corrected version of Duporcq's theorem

Based on Translation 4 and on the results of Section 4.1, we give the following corrected version of Duporcq's theorem:

Theorem 5 If five pairs of anchor points (m*_i_*,M*_i_*) with (m*_i_*,M*_i_*) ≠ (m*_j_*,M*_j_*) for *i* ≠ *j* and *i*,*j* ∈ {1,…,5} of a planar manipulator are given, then there exists – with exception of one case – a sixth point pair (m_6_,M_6_) with (m_6_,M_6_) ≠ (m_k_,M_k_) *for k* = 1,…,5 in a way that the resulting planar architecturally singular manipulator has the same solution for the direct kinematics as the given one, where the pair (m*_l_*,M*_l_*) for *l* = 1,…,6 fulfills the

• sphere condition, if m*_l_* and M*_l_* are finite,
• Darboux condition, if m*_l_* is finite and M*_l_* is an ideal point,
• Mannheim condition, if m*_l_* is an ideal point and M*_l_* is finite,
• angle condition, if m*_l_* and M*_l_* are ideal points.

The only exception, where the point pair (m_6_,M_6_) does not exist, is if the given five point pairs fulfill the following conditions:

(a) m_1_,…,m_5_ as well as M_1_,…,M_5_ are pairwise distinct.
(b) There does not exist a triple of point pairs that m*_i_*,m*_j_*,m*_k_* and M*_i_*,M*_j_*,M*_k_* are both collinear for pairwise distinct *i*,*j*,*k* ∈ {1,…,5}.
(c) No four points of {m_1_,…,m_5_} or {M_1_,…,M_5_} are located on a line.
(d) The following relation of the cross-ratios of lines^8^:
  (1) $$\mathrm{CR}\left(g_{i,j},g_{i,k},g_{i,l},g_{i,m}\right)=\mathrm{CR}\left(G_{i,j},G_{i,k},G_{i,l},G_{i,m}\right)$$
  is fulfilled for only one *i* ∈ {1, …,5}, where the indices *j*, *k*, *l*, *m* are pairwise distinct with *j*, *k*, *l*, *m* ∈ {1, …,5} \ {*i*}. Moreover, g_*i*,*j*_ (resp. G_*i*,*j*_) denotes the connecting line of m_*i*_ and m_*j*_ (resp. M_*i*_ and M_*j*_).

The point pair (m_6_,M_6_) is uniquely defined if items (a), (b), (c) hold and if the platform anchor points m_1_, …, m_5_ and the base anchor points M_1_, …, M_5_ are not coupled by a projectivity. In all other cases, there exist at least a one-parametric set of solutions for the point pair (m_6_,M_6_).

We subdivide the proof of this theorem into three parts.

### 5.1 Preparatory considerations

In this part, we want to reason the conditions given in items (a), (b), (c):

ad (a) If e.g. M_1_ = M_2_ holds, then there exists a one-parametric solution for (m_6_,M_6_) with M_6_ = M_1_ = M_2_ and m_6_ ∈ g_1,2_ (cf. item 6 of Theorem 3 of [19]). Note that the line g_1,2_ is uniquely determined, as (m_1_,M_1_) ≠ (m_2_,M_2_) has to hold. Clearly, these considerations can be done for any two base or platform anchor points, which already yields the result. Therefore, we can assume for the remainder of this proof that item (a) holds.
ad (b) If m*_i_*,m*_j_*,m*_k_* are located on a line g_*i*,*j*,*k*_ and if M*_i_*,M*_j_*,M*_k_* are located on a line G_*i*,*j*,*k*_ for *i* ≠ *j* ≠ *k* ≠ *i* and *i*, *j*, *k* ∈ {1, …,5}, then there exists again a one-parametric solution for (m_6_,M_6_) with m_6_ ∈ g_*i*,*j*,*k*_, M_6_ ∈ G_*i*,*j*,*k*_ and *CR*(m_*i*_,m_*j*_,m_*k*_,m_6_) = CR(M_*i*_,M_*j*_,M_*k*_,M_6_) (cf. page 222 of [29] or item 8 of Theorem 3 of [19]). Therefore, we can assume for the remainder of this proof that item (b) holds.
ad (c) If e.g. m_1_, m_2_, m_3_, m_4_ are collinear, then there exists a one-parametric solution for (m_6_,M_6_) with m_6_ ∈ g_1,2,3,4_ in a way that there is a projective correspondence between the points of *g*_1,2,3,4,6_ and the points of the conic section defined by M_1_, M_2_, M_3_, M_4_, M_6_ (cf. item 10 of Theorem 3 of [19], [30], [31]). Therefore, we can assume for the remainder of this proof that item (c) holds.

Remark 5 The one-parametric solution set of item (a) is a pencil of lines (ruled surface of degree 1). The one-parametric solution set of item (b) and item (c) results in flexible ruled surfaces of degree 2 and 3, respectively. Moreover in this context it should be noted that the one-parametric solution set of Theorem 2 yields flexible ruled surfaces of degree 4. ⋄

Lemma 1 There do not exist architecturally singular manipulators with four points collinear, which fulfill the assumptions (a), (b), (c) of Theorem 5.

Proof An architecturally singular manipulator with four collinear points, can only be one of the 12 designs listed by Karger in Theorem 3 of [19]. First of all, we can exclude all designs, where at least five anchor points are collinear, as they contradict item (c). These are the items 1,3,4,5,7 and 10.

Moreover, due to item (a), manipulators are allowed to have maximally two pairs of coinciding anchor points, namely M_6_ = M_*i*_ and m_6_ = m_*j*_ with *i* ≠ *j* and *i*, *j* ∈ {1, …,5}. This already excludes the items 2,6,9 and 11.

Therefore, we remain only with two cases. In item 12 we have M_5_ = M_6_ and m_1_, …, m_4_ collinear, which contradicts assumption (c). For case 8 assumption (b) yields the contradiction. □

Now we proceed with the proof of Theorem 5. Due to Lemma 1, we can assume for the remaining part of this proof, that no four platform anchor points m_1_, …, m_6_ or base anchor points M_1_, …, M_6_ are collinear. This implies together with Lemma 1 of [12], that the given 5-legged manipulator cannot be a degenerated one (cf. Remark 1). On the other hand, for each degenerated 5-legged manipulator at least a one-parametric solution for (m_6_,M_6_) exists according to the above discussed three cases (a), (b) and (c).

Moreover it should be noted, that Lemma 1 also closes Gap 3 of Section 2.1.^9^ In the following, we investigate the last remaining gap, where we distinguish a general case and a special one.

### 5.2 Proof of the general case

In this case, we assume that m_1_, m_2_, m_3_, m_4_ form a quadrilateral as well as the corresponding base anchor points M_1_, M_2_, M_3_, M_4_. We apply projectivities to the platform and the base in a way that the anchor points have the following projective coordinates in the planar platform and the planar base, respectively:

$$\begin{array}{rr} M_{1}=m_{1}={\left(1:0:0\right)}^{T}, & M_{2}=m_{2}={\left(0:1:0\right)}^{T},\\ M_{3}=m_{3}={\left(0:0:1\right)}^{T}, & M_{4}=m_{4}={\left(1:1:1\right)}^{T}. \end{array}$$

Moreover, we denote the homogeneous coordinates of M_5_ and m_5_ by:

$$\begin{array}{cc} M_{5}={\left(x_{0}:x_{1}:x_{2}\right)}^{T}, & m_{5}={\left(y_{0}:y_{1}:y_{2}\right)}^{T}. \end{array}$$

Now, we have exactly the same situation as in Section 3 of [22].^10^ The quadrilaterals m_1_, m_2_, m_3_, m_4_ and M_1_, M_2_, M_3_, M_4_ determine a non-singular projectivity *κ* from the platform to the base. If m_5_*κ* = M_5_ holds, we end up with the special case given in Theorem 2, which is known to have a one-parametric solution.

Therefore, we can assume that m_5_*κ* ≠ M_5_ holds. In this case, it was shown in Section 3 of [22], that a unique sixth point pair (m_6_,M_6_) exists with the following homogeneous coordinates:

$$\begin{array}{l} M_{6}=\left(\begin{array}{c} X_{0}\\ X_{1}\\ X_{2} \end{array}\right)\mathbb{R}:=\left(\begin{array}{c} \left(y_{2}-y_{1}\right)\left(y_{1}x_{0}-y_{0}x_{1}\right)\left(y_{2}x_{0}-y_{0}x_{2}\right)\\ \left(y_{2}-y_{0}\right)\left(y_{1}x_{0}-y_{0}x_{1}\right)\left(y_{2}x_{1}-y_{1}x_{2}\right)\\ \left(y_{1}-y_{0}\right)\left(y_{2}x_{0}-y_{0}x_{2}\right)\left(y_{2}x_{1}-y_{1}x_{2}\right) \end{array}\right)\mathbb{R},\\ m_{6}=\left(\begin{array}{c} Y_{0}\\ Y_{1}\\ Y_{2} \end{array}\right)\mathbb{R}:=\left(\begin{array}{c} \left(x_{2}-x_{1}\right)\left(y_{1}x_{0}-y_{0}x_{1}\right)\left(y_{2}x_{0}-y_{0}x_{2}\right)\\ \left(x_{2}-x_{0}\right)\left(y_{1}x_{0}-y_{0}x_{1}\right)\left(y_{2}x_{1}-y_{1}x_{2}\right)\\ \left(x_{1}-x_{0}\right)\left(y_{2}x_{0}-y_{0}x_{2}\right)\left(y_{2}x_{1}-y_{1}x_{2}\right) \end{array}\right)\mathbb{R}. \end{array}$$

According to Gap 2 of Section 2.1, we have to check if (m_6_,M_6_) can coincide with one of the first five point pairs.

Case (m_6_,M_6_) = (m_*i*_,M_*i*_) for *i* ∈ {1,2,3,4}:

By solving the corresponding system of equations^11^ it can be verified without difficulties, that this can only be the case, if the line g_i,5_ is mapped under *κ* on the line G_i,5_. Under consideration of some fundamentals of projective geometry, this fact can also be rewritten as: *CR*(g_*i*,*j*_,g_*i*,*k*_,g_*i*,*l*_,g_*i*,5_) = *CR*(G_*i*,*j*_,G_*i*,*k*_,G_*i*,*l*_,G_*j*,5_) for pairwise distinct *i*, *j*, *k*, *l* ∈ {1,2,3,4}.

As the discussion of the remaining case is a little different, it is done separately within the next paragraph.

Case (m_6_,M_6_) = (m_5_,M_5_):

In this case, the corresponding system of equations

$$\begin{array}{rrrr} X_{0}x_{1}-X_{1}x_{0}=0, & X_{0}x_{2}-X_{2}x_{0}=0, & Y_{0}y_{1}-Y_{1}y_{0}=0, & Y_{0}y_{2}-Y_{2}y_{0}=0, \end{array}$$

can only have a solution if the following condition holds:

$$x_{0}x_{1}\left(y_{0}y_{2}-y_{1}y_{2}\right)+x_{0}x_{2}\left(y_{1}y_{2}-y_{0}y_{1}\right)+x_{1}x_{2}\left(y_{0}y_{1}-y_{0}y_{2}\right)=0.$$

Assume that m_5_ is given, then this equation corresponds with the geometric condition that M_5_ is located on the conic section defined by M_1_, M_2_, M_3_, M_4_ and m_5_*κ*. This is equivalent with the statement that the conic section defined by m_1_, …, m_5_ is mapped under *κ* on the conic section defined by M_1_, …, M_5_. Under consideration of Jakob Steiner's generation of a conic, as the intersection points of corresponding lines of two projectively related pencils of lines with distinct centers, the above result can be rewritten in terms of cross-ratios of lines as follows: *CR*(g_5,1_,g_5,2_,g_5,3_,g_5,4_) = *CR*(G_5,1_,G_5,2_,G_5,3_,G_5,4_).

Therefore, all exceptional cases (m_6_,M_6_) = (m_*i*_,M_*i*_) for *i* ∈ {1,2,3,4,5} are projectively equivalent and can be written in the unified way of Eq. (1).

Remark 6 Within the frame of Duporcq's argumentation, the case (6,6′) = (i,i′) for i ∈ {1, …,5} can occur, either if *Γ*_3_ and *Γ*_4_ touch each other in the point i, or if i is a singular point of *Γ*_3_ or *Γ*_4_. ⋄

If for two different *i* ∈ {1, …,5} Eq. (1) is fulfilled, then it follows immediately from the above given geometric interpretation (under consideration of the assumptions (a), (b) and (c) of Section 5.1), that M_5_ = m_5_*κ* has to hold; a contradiction.

In order to exclude all possibility of doubt, we finally check, whether it is possible that M_6_ or m_6_ are not defined, i.e. (*X*_0_ : *X*_1_ : *X*_2_) = (0 : 0 : 0) and (*Y*_0_ : *Y*_1_ : *Y*_2_) = (0 : 0 : 0), respectively. It can easily be verified by direct computations, that both cases cannot appear under our assumptions. This finishes the proof of the general case.

### 5.3 Proof of the special case

**Fig. 6:**
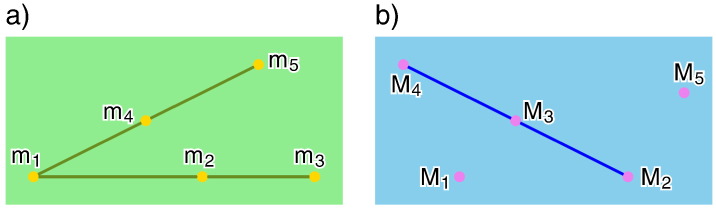
Special case: a) Sketch of the platform. b) Sketch of the base.
• M_2_, M_3_, M_4_ are collinear (cf. Fig. 6b).

Due to Section 5.2 we can assume that there do not exist four pairs of anchor points (m_*i*_,M_*i*_), (m_*j*_,M_*j*_), (m_*k*_,M_*k*_), (m_*l*_,M_*l*_) with pairwise distinct *i*, *j*, *k*, *l* ∈ {1,2,3,4,5}, that m_*i*_, m_*j*_, m_*k*_, m_*l*_ as well as M_*i*_, M_*j*_, M_*k*_, M_*l*_ form quadrilaterals. It is not difficult to verify within a short study of cases, that this is only possible if the following conditions are fulfilled (after may a necessary renumbering of indices and exchange of the platform and base):

• m_1_, m_2_, m_3_ are collinear and m_1_, m_4_, m_5_ are collinear (cf. Fig. 6a),

In the next step, we show that there does not exist a sixth point pair if these three conditions hold. This is done by contradiction, i.e. we assume that (m_6_,M_6_) exists and construct a contradiction. Due to our assumptions (a), (b), (c) and Lemma 1, we can apply Lemma 2 of Karger [18] without loss of generality. This yields that also the points M_4_, M_5_, M_6_ are collinear, M_2_, M_3_, M_6_ are collinear and m_1_, m_5_, m_6_ are collinear. As a consequence, the points M_2_, …, M_6_ as well as m_1_, m_4_, m_5_, m_6_ have to be located on a line, which contradicts assumption (c).

As for this special case g_4,1_ = g_4,5_ holds, the cross-ratio *CR*(g_4,1_,g_4,2_,g_4,3_,g_4,5_) equals infinity (cf. Appendix). The same is true for *CR*(G_4,1_,G_4,2_,G_4,3_,G_4,5_) as G_4,2_ = G_4,3_ holds. Therefore, this special case is also contained within the formulation used in Theorem 5.

Finally, we have to check what happens if Eq. (1) is additionally fulfilled for another *i* ∈ {1,2,3,5}. This can only be the case if and only if m_*i*_, m_*j*_, m_*k*_ are collinear (⇔ g_*i*,*j*_ = g_*i*,*k*_) and M_*i*_, M_*l*_, M_*m*_ are collinear (⇔ G_*i*,*l*_ = G_*i*,*m*_) for pairwise distinct *j*, *k*, *l*, *m* ∈ {1, …,5} \ {*i*}. It can easily be seen that for each *i* ∈ {1,2,3,5}, we end up with a contradiction with respect to the assumptions of Section 5.1. This finishes the proof of Theorem 5. □

Theorem 5 can be seen as the corrected version of Translation 4 of Duporcq's theorem. By giving the following example, we show that Theorem 5 cannot be generalized in the sense of Translation 5 (cf. Remark 2).

Remark 7 Theorem 5 also corrects the statement of Wohlhart in Section 4 of [24] (cf. Remark 3), that there is “only one non-trivial solution” for the sixth pair of anchor points. ⋄

## 6 Example

**Fig. 7:**
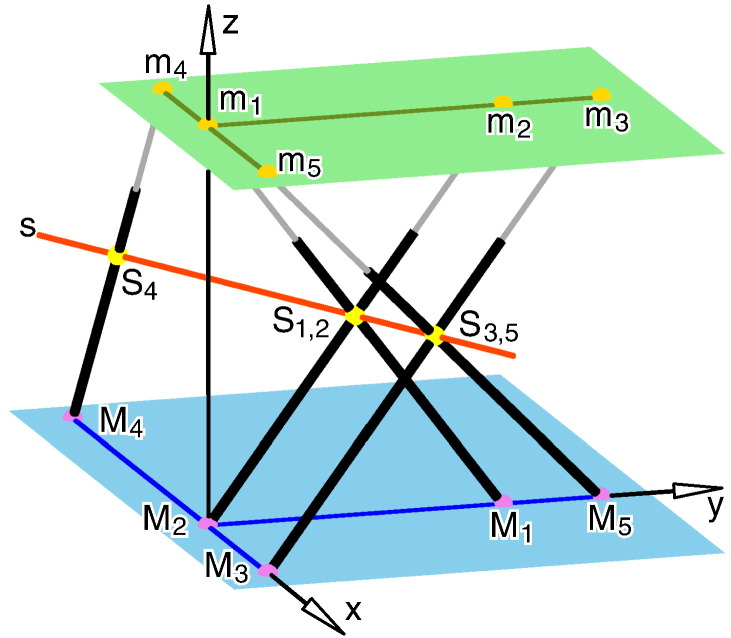
Axonometric view of the 5-legged manipulator in its initial pose.

In the initial pose of the 5-legged manipulator, the coordinates of the anchor points with respect to the fixed frame (x,y,z) are given by (cf. Fig. 7):

$$\begin{array}{ccc} M_{1}={\left(030\right)}^{T}, & m_{1}={\left(004\right)}^{T}, & R_{1}=∥M_{1}-m_{1}∥=5,\\ M_{2}={\left(000\right)}^{T}, & m_{2}={\left(034\right)}^{T}, & R_{2}=∥M_{2}-m_{2}∥=5,\\ M_{3}={\left(200\right)}^{T}, & m_{3}={\left(044\right)}^{T}, & R_{3}=∥M_{3}-m_{3}∥=6,\\ M_{4}={\left(-9/2,0,0\right)}^{T}, & m_{4}={\left(-3/2,0,4\right)}^{T}, & R_{4}=∥M_{4}-m_{4}∥=5,\\ M_{5}={\left(040\right)}^{T}, & m_{5}={\left(204\right)}^{T}, & R_{5}=∥M_{5}-m_{5}∥=6. \end{array}$$

As the geometry of this example fulfills the conditions of the special case (cf. Fig. 6), there does not exist a sixth pair of anchor points, that the resulting manipulator is architecturally singular.

Moreover, it can easily be checked, that in the given pose the Plücker coordinates of the carrier lines l_i_ of the five legs $\bar{m_{i}M_{i}}$ (for *i* = 1, …, 5) are linearly independent. Therefore, these five lines l_1_, …, l_5_ determine uniquely a linear line complex C. In our case, C is even a singular one (cf. footnote 5), as all lines l_i_ intersect the axis s of C in a point S_i_ (cf. Fig. 7). Note that the parametric expression **s** of the axis s can be given by:

(2) $$s={\left(2t-3,t,2\right)}^{T}\;\mathrm{for}\;t\in \mathbb{R}.$$

### 6.1 Direct kinematics of the 5-legged manipulator

For solving the direct kinematics, it is advantageous to work in the Study parameter space *P*_ℝ_^7^, which is a 7-dimensional real projective space with homogeneous coordinates *e*_0_ : … : *e*_3_ : *f*_0_ : … : *f*_3_. By using these so-called Study parameters for the parametrization of Euclidean displacements, the coordinates **m**_*i*_′ of the platform anchor points with respect to *Σ*_0_ can be written as *K***m**_*i*_′ = **Rm**_*i*_ + (*t*_1_,*t*_2_,*t*_3_)^*T*^ with

$$\begin{array}{l} t_{1}:=2\left(e_{0}f_{1}-e_{1}f_{0}+e_{2}f_{3}-e_{3}f_{2}\right),t_{2}:=2\left(e_{0}f_{2}-e_{2}f_{0}+e_{3}f_{1}-e_{1}f_{3}\right),\\ t_{3}:=2\left(e_{0}f_{3}-e_{3}f_{0}+e_{1}f_{2}-e_{2}f_{1}\right), \end{array}$$

and the rotational matrix

$$R:=\left(r_{\mathrm{ij}}\right)=\left(\begin{array}{ccc} e_{0}^{2}+e_{1}^{2}-e_{2}^{2}-e_{3}^{2} & 2\left(e_{1}e_{2}-e_{0}e_{3}\right) & 2\left(e_{1}e_{3}+e_{0}e_{2}\right)\\ 2\left(e_{1}e_{2}+e_{0}e_{3}\right) & e_{0}^{2}-e_{1}^{2}+e_{2}^{2}-e_{3}^{2} & 2\left(e_{2}e_{3}-e_{0}e_{1}\right)\\ 2\left(e_{1}e_{3}-e_{0}e_{2}\right) & 2\left(e_{2}e_{3}+e_{0}e_{1}\right) & e_{0}^{2}-e_{1}^{2}-e_{2}^{2}+e_{3}^{2} \end{array}\right),$$

with the Euler parameters *e*_0_, …, *e*_3_. Now, all points of *P*_*ℝ*_^7^, which are located on the so-called Study quadric *Φ* : ∑ _*i* = 0_^3^*e*_*i*_*f*_*i*_ = 0, correspond with Euclidean displacements with exception of the subspace *e*_0_ = … = *e*_3_ = 0, as these points cannot fulfill the normalizing condition *K* = 1 with *K* := *e*_0_^2^ + *e*_1_^2^ + *e*_2_^2^ + *e*_3_^2^.

Husty [32] showed, that the condition for m_i_ to be located on a sphere with center M_i_ and radius *R*_*i*_ can be expressed by a homogeneous quadratic equation *Λ*_*i*_ in the Study parameters. For the explicit expression of *Λ*_*i*_ we refer to Eq. (14) of [32]. Therefore, the solution for the direct kinematics can be determined as the algebraic variety $V_{\Lambda }$ of the ideal *ℐ*_*Λ*_ spanned by *Φ*, Δ_2_, …, Δ_5_, *Λ*_1_ with Δ_*j*_ := *Λ*_*j*_ − *Λ*_1_. As the polynomials Δ_*j*_ are only linear in *f*_0_, …, *f*_3_, we can solve *Φ*, Δ_*i*_, Δ_*j*_, Δ_*k*_ for *f*_0_, …, *f*_3_ and plug the resulting expressions into Δ_*l*_ and *Λ*_1_. Then we eliminate *e*_3_ by computing the resultant *Ξ*_*l*_ of Δ_*l*_ and *Λ*_1_. As this can be done in four ways for the pairwise distinct indices *i*, *j*, *k*, *l* ∈ {2,3,4,5}, the greatest common divisor *ϒ*_*Λ*_ of *Ξ*_2_, …, *Ξ*_5_ corresponds to the solution of the forward kinematics. For our given example *ϒ*_*Λ*_ is a homogeneous polynomial in *e*_0_, *e*_1_, *e*_2_ of degree 12:

(3) $${ϒ}_{\Lambda }:k_{10}e_{0}^{10}+k_{8}e_{0}^{8}+k_{6}e_{0}^{6}+k_{4}e_{0}^{4}+k_{2}e_{0}^{2}+k_{0}=0,$$

where the explicit expressions for *k*_0_, …, *k*_10_ are given in the Appendix.

Now, we check if there exists a sixth point pair (m_6_,M_6_), that the resulting non-architecturally singular manipulator has the same solution for the direct kinematics as the given 5-legged one. For this purpose, we have to distinguish the following four cases.

### 6.2 (m_6_,M_6_) determine a sphere condition

In this case, m_6_ and M_6_ are finite points. Moreover, the carrier line l_6_ of the sixth leg $\bar{m_{6}M_{6}}$ also has to intersect s in the initial pose of Fig. 7. Under consideration of Eq. (2), this constraint implies **m**_6_ = (4*t* − 6 − *x*, 2*t* − *y*, 4)^*T*^ and

$$R_{6}=2\sqrt{13+6x+x^{2}+y^{2}+t\left(5t-12-4x-2y\right)},$$

for **M**_6_ = (*x*,*y*,0)^*T*^. Therefore, we are only left with the three unknowns *x*, *y*, *t*. Moreover, we can also compute *Λ*_6_(*x*,*y*,*t*) according to Eq. (14) of [32] and Δ_6_(*x*,*y*,*t*).

Now, the solution of the direct kinematics of the SG platform is given by the algebraic variety of the ideal spanned by *Φ*, *Λ*_1_, Δ_2_, …, Δ_6_(*x*,*y*,*t*). In our case, this variety has to equal the one-dimensional variety $V_{\Lambda }$ of Section 6.1. A necessary condition for this circumstance is, that the variety $V_{\Delta }$ of the subideal *ℐ*_Δ_ spanned by *Φ*, Δ_2_, …, Δ_6_(*x*,*y*,*t*) contains (or equals) $V_{\Lambda }$. This is checked in the remainder of this section.

After replacing *Λ*_1_ by Δ_6_(*x*,*y*,*t*), the elimination of the unknowns within the ideal *ℐ*_Δ_, can be done as for the ideal *ℐ*_*Λ*_ (cf. Section 6.1). In this case, we end up with the polynomial *ϒ*_*Δ*_ of the form:

(4) $${ϒ}_{\Delta }:u_{\Delta }e_{0}^{2}+v_{\Delta }=0,$$

where *u*_*Δ*_ and *v*_*Δ*_, which are given in the Appendix, are homogeneous polynomials in *e*_1_, *e*_2_ of degrees 2 and 4, respectively. Now it can be seen at the basis of the degrees of *ϒ*_Δ_ and *ϒ*_*Λ*_ with respect to *e*_0_, *e*_1_, *e*_2_, that the zero set of *ϒ*_*Λ*_ cannot be contained within the zero set of *ϒ*_Δ_. Therefore, we only remain with the possibility that *u*_Δ_*e*_0_^2^ + *v*_Δ_ = 0 is fulfilled for all *e*_0_, *e*_1_, *e*_2_. It can easily be seen by a simple case study, that the resulting system of equations in *x*, *y*, *t* has the following five solutions: (m_6_,M_6_) = (m_i_,M_i_) for *i* ∈ {1, …,5}.

Remark 8 Note that for *i* = 4, we get a double solution, which confirms that this example is also a counter example to Duporcq's theorem in the sense of Gap 2 of Section 2.1. ⋄

### 6.3 (m_6_,M_6_) determine a Darboux condition

In this case, M_6_ is an ideal point and m_6_ is finite. In the following we consider the initial pose given in Fig. 7: If M_6_ differs from the ideal point of the axis s, then the connecting line of M_6_ with any finite point of s is always parallel to the platform. This yields a contradiction, as this implies that m_6_ has to be an ideal point as well. Therefore, M_6_ equals the ideal point of s and m_6_ is any finite point with coordinates **m**_6_ = (*x*,*y*,4)^*T*^.

Now, the Darboux condition *Ω*_6_ can be computed according to [12], which yields:

$$\begin{array}{l} \Omega_{6}:x\left(e_{0}e_{3}+e_{1}e_{2}-2e_{2}^{2}-2e_{3}^{2}\right)+y\left(2e_{1}e_{2}-2e_{0}e_{3}-e_{1}^{2}-e_{3}^{2}\right)+\\ 4\left(2e_{1}e_{3}+2e_{0}e_{2}+e_{2}e_{3}-e_{0}e_{1}\right)+2\left(e_{0}f_{1}-e_{1}f_{0}+e_{2}f_{3}-e_{3}f_{2}\right)+\\ e_{0}f_{2}-e_{2}f_{0}+e_{3}f_{1}-e_{1}f_{3}=0. \end{array}$$

Analogous considerations as in Section 6.2 yield for the ideal *ℐ*_*Ω*_ spanned by *Φ*, *Δ*_2_, …, *Δ*_5_, *Ω*_6_(*x*,*y*) the expression:

(5) $${ϒ}_{\Omega }:u_{\Omega }e_{0}^{2}+v_{\Omega }=0,$$

where *u*_*Ω*_ and *v*_*Ω*_ are given in the Appendix. For the same reason as in Section 6.2, the equation *ϒ*_*Ω*_ has to be fulfilled identically. It can easily be checked, that the resulting system of equations in *x*, *y* does not possess a solution.

### 6.4 (m_6_,M_6_) determine a Mannheim condition

Similar considerations as in Section 6.3 for the initial pose show, that m_6_ has to be the ideal point of the axis s. By denoting the coordinates of the finite point M_6_ by **M**_6_ = (*x*,*y*,0)^*T*^, the Mannheim condition *Π*_6_ can be computed according to [12], which yields:

$$\begin{array}{l} \Pi_{6}:x\left(e_{1}e_{2}-e_{0}e_{3}-2e_{2}^{2}-2e_{3}^{2}\right)+y\left(2e_{0}e_{3}+2e_{1}e_{2}-e_{3}^{2}-e_{1}^{2}\right)+\\ 2\left(e_{1}f_{0}-e_{0}f_{1}+e_{2}f_{3}-e_{3}f_{2}\right)-e_{0}f_{2}+e_{2}f_{0}+e_{3}f_{1}-e_{1}f_{3}=0. \end{array}$$

Now the ideal *ℐ*_*Π*_ spanned by *Φ*, Δ_2_, …, Δ_5_, *Π*_6_(*x*,*y*) implies:

(6) $${ϒ}_{\Pi }:u_{\Pi }e_{0}^{2}+v_{\Pi }=0,$$

where *v*_*Π*_ equals *v*_*Ω*_ and *u*_*Π*_ is given in the Appendix. For the same reason as in Section 6.2, the equation *ϒ*_*Π*_ has to be fulfilled identically. Again, it can easily be verified, that the resulting system of equations in *x*, *y* does not possess a solution.

### 6.5 (m_6_,M_6_) determine an angle condition

In this case, m_6_ and M_6_ are both ideal points. m_6_ is the ideal point in direction (*x*,*y*,0) ≠ (0,0,0) and M_6_ is the ideal point in direction (*X*,*Y*,0) ≠ (0,0,0). Therefore, the angle condition ∢ _6_ can be computed as:

$${∢}_{6}:\left[R{\left(x,y,0\right)}^{T}\right]\cdot {\left(X,Y,0\right)}^{T}-\left[{\left(x,y,0\right)}^{T}\cdot {\left(X,Y,0\right)}^{T}\right]K=0.$$

Now the ideal *ℐ*_∢_ spanned by *Φ*, Δ_2_, …, Δ_5_, ∢ _6_(*x*,*y*,*X*,*Y*) implies:

(7) $${ϒ}_{∢}:u_{∢}e_{0}^{2}+v_{∢}=0,$$

where *u*_∢_ and *v*_∢_ are given in the Appendix. For the same reason as in Section 6.2, the equation *ϒ*_∢_ has to be fulfilled identically. Also in this case it can be checked without difficulties, that the resulting system of equations in *x*, *y*, *X*, *Y* does not possess a solution.

This finishes all possible cases. Therefore, we have demonstrated on basis of the given example that Theorem 5 cannot be generalized in the sense of Translation 5. This result also closes the study on Duporcq's theorem.

## Appendix

Cross-ratio:

The cross-ratio of four points A, B, C, D of a line g is given:

• for B ≠ C and A ≠ D by:
  $$\mathrm{CR}\left(A,B,C,D\right):=\frac{\left|\begin{array}{cc} a_{0} & c_{0}\\ a_{1} & c_{1} \end{array}\right|\cdot \left|\begin{array}{cc} b_{0} & d_{0}\\ b_{1} & d_{1} \end{array}\right|}{\left|\begin{array}{cc} b_{0} & c_{0}\\ b_{1} & c_{1} \end{array}\right|\cdot \left|\begin{array}{cc} a_{0} & d_{0}\\ a_{1} & d_{1} \end{array}\right|},$$
• for B = C or A = D by: *CR*(A,B,C,D) := *∞*,

where (*a*_0_ : *a*_1_), …, (*d*_0_ : *d*_1_) are the homogeneous coordinates of the points A, …, D with respect to g (cf. page 20 of [10]).

The cross-ratio *CR*(a,b,c,d) of four lines a, b, c, d through a common point P can be computed as given above, if (*a*_0_ : *a*_1_), …, (*d*_0_ : *d*_1_) are the homogeneous coordinates of the lines a, …, d with respect to the pencil of lines through P.

Coefficients of Eqs. (3), (4), (5), (6), (7):

$$\begin{array}{l} k_{10}:=262144{\left(e_{1}-2e_{2}\right)}^{2},\\ k_{8}:=1381872e_{2}^{4}+493663e_{1}^{4}-7334680e_{1}^{2}e_{2}^{2}+7224352e_{2}^{3}e_{1}-1395960e_{1}^{3}e_{2},\\ k_{6}:=4\left(658443e_{2}e_{1}^{5}-7337412e_{2}^{2}e_{1}^{4}+3830132e_{2}^{5}e_{1}-4410487e_{2}^{3}e_{1}^{3}-374784e_{2}^{6}+3051260e_{2}^{4}e_{1}^{2}+36575e_{1}^{6}\right),\\ k_{4}:=2\left(1915306e_{1}^{7}e_{2}-1527280e_{2}^{8}-7274462e_{1}^{6}e_{2}^{2}+5585170e_{2}^{6}e_{1}^{2}-43306426e_{2}^{3}e_{1}^{5}+4446192e_{2}^{7}e_{1}-49034108e_{2}^{4}e_{1}^{4}+33089240e_{2}^{5}e_{1}^{3}-73187e_{1}^{8}\right),\\ k_{2}:=4\left(803542e_{1}^{8}e_{2}^{2}-40837984e_{1}^{5}e_{2}^{5}+221841e_{1}^{9}e_{2}-5258480e_{1}^{2}e_{2}^{8}-331776e_{2}^{10}+11499410e_{1}^{4}e_{2}^{6}-41012864e_{1}^{6}e_{2}^{4}-16929e_{1}^{10}+19445900e_{1}^{3}e_{2}^{7}-8824193e_{1}^{7}e_{2}^{3}+1021068e_{1}e_{2}^{9}\right),\\ k_{0}:={\left(324e_{2}^{6}-3456e_{2}^{5}e_{1}+1720e_{2}^{4}e_{1}^{2}+10906e_{2}^{3}e_{1}^{3}+4736e_{2}^{2}e_{1}^{4}+234e_{2}e_{1}^{5}-81e_{1}^{6}\right)}^{2},\\ u_{\Delta }:=4\left(8x^{2}+8y^{2}+72t+27y-34\mathrm{yt}+12x-23\mathrm{xt}-108\right)\left(8x^{2}e_{2}^{2}+128xe_{1}e_{2}-16\mathrm{xy}e_{1}e_{2}-23\mathrm{xt}e_{2}^{2}-16\mathrm{xt}e_{1}e_{2}+12xe_{2}^{2}-144ye_{1}e_{2}-16\mathrm{yt}e_{1}^{2}-384te_{2}e_{1}+27ye_{2}^{2}-18\mathrm{yt}e_{2}^{2}+96\mathrm{yt}e_{1}e_{2}+576e_{1}e_{2}+72te_{2}^{2}-108e_{2}^{2}+8y^{2}e_{1}^{2}\right),\\ v_{\Delta }:={\left(216e_{1}e_{2}-144te_{1}e_{2}-18\mathrm{yt}e_{1}^{2}+18\mathrm{yt}e_{2}^{2}-28\mathrm{yt}e_{1}e_{2}+32x^{2}e_{1}e_{2}+32y^{2}e_{1}e_{2}-110\mathrm{xt}e_{1}e_{2}-54ye_{1}e_{2}-18\mathrm{xy}e_{1}e_{2}-9y^{2}e_{2}^{2}+192xe_{1}e_{2}+9y^{2}e_{1}^{2}\right)}^{2},\\ u_{\Omega }:=4\left(2y-72-41x\right)\left(48xe_{1}e_{2}-41xe_{2}^{2}-72e_{2}^{2}-16ye_{1}^{2}-32ye_{1}e_{2}+128e_{1}e_{2}+18ye_{2}^{2}\right),\\ v_{\Omega }:=4{\left(55xe_{1}e_{2}+9ye_{1}^{2}+14ye_{1}e_{2}+72e_{1}e_{2}-9ye_{2}^{2}\right)}^{2},\\ u_{\Pi }:=4\left(23x+34y-72\right)\left(16xe_{1}e_{2}+23xe_{2}^{2}+16ye_{1}^{2}+18ye_{2}^{2}+384e_{1}e_{2}-72e_{2}^{2}-96ye_{1}e_{2}\right),\\ u_{∢}:=4\left(16\mathrm{yY}+9\mathrm{xY}-9\mathrm{yX}+16\mathrm{xX}\right)\left(16\mathrm{xX}e_{2}^{2}+9\mathrm{xY}e_{2}^{2}-48\mathrm{xY}e_{1}e_{2}-9\mathrm{yX}e_{2}^{2}+16\mathrm{yX}e_{1}e_{2}+16\mathrm{yY}e_{1}^{2}\right),\\ v_{∢}:=4{\left(-9\mathrm{yX}e_{1}e_{2}+32\mathrm{yY}e_{1}e_{2}+32\mathrm{xX}e_{1}e_{2}-9\mathrm{xY}e_{1}e_{2}+9\mathrm{yY}e_{1}^{2}-9\mathrm{yY}e_{2}^{2}\right)}^{2}. \end{array}$$

## Footnotes

2 Bricard cited Duporcq's theorem in item VII of his introduction to [7].

3 The content of this paper was reprinted in a slightly different version within the frame of Duporcq's book [9], which was also written in French.

5 The geometric interpretation of the degenerated planar cases, as well as sufficient conditions for them, were given by the author in [20].

6 A linear line complex C is called singular, if all lines of this set intersect a common line, which is the so-called axis of C (cf. page 159 ff. of [10]).

7 Clearly, in this way examples can be constructed as well, where only M_6_*μ*′ is an ideal point or both points m6*μ* and M_6_*μ*′ are at infinity.

9 With respect to Gap 3, Duporcq was lucky, as it does not cause any counter example.

10 It can easily be seen, that we can always relabel the first four point pairs that neither M_1_, M_4_, M_5_ nor m_2_, m_3_, m_5_ are collinear (cf. footnote 1 of [22]).

11 E.g. for *i* = 1 the set of equations is given by *X*_1_ = 0, *X*_2_ = 0, *Y*_1_ = 0, *Y*_2_ = 0, which implies the solution *x*_1_ : *x*_2_ = *y*_1_ : *y*_2_.

## Footnotes

4 Note that in the terms of contemporary geometry, the points *α*, *β* and *γ* are the singular points of this transformation.

8 For the definition of the cross-ratio of points and lines please see the Appendix.

## References

[1] Duporcq E. (1898). Sur la correspondance quadratique et rationnelle de deux figures planes et sur un déplacement remarquable. C. R. Seances Acad. Sci..

[2] Emch A., Snyder V. (1928). Selected Topics in Algebraic Geometry.

[3] Forder H.G. (1960).

[4] Husty M., Ceccarelli M. (2000). International Symposium on History of Machines and Mechanisms.

[5] Karger A. (2010). Parallel manipulators and Borel–Bricard's problem. Comput. Aided Geom. Des..

[6] Borel E. (1908). Mémoire sur les déplacements à trajectoires sphériques. Mém. Présent. Var. Sci. Acad. Sci. Natl. Inst. Fr..

[7] Bricard R. (1906). Mémoire sur les déplacements à trajectoires sphériques. J. École Polytech..

[8] Nawratil G. (2012). Review and recent results on Stewart Gough platforms with self-motions. Appl. Mech. Mater..

[9] Duporcq E. (1899).

[10] Pottmann H., Wallner J. (2001).

[11] Chasles M. (1861). Sur les six droites qui peuvent étre les directions de six forces en équilibre. C. R. Seances Acad. Sci..

[12] Nawratil G. (2013). Types of self-motions of planar Stewart Gough platforms. Meccanica.

[13] Merlet J.-P. (1992). Singular configurations of parallel manipulators and Grassmann geometry. Int. J. Robot. Res..

[14] Karger A., Lencarci J., Husty M.L. (1998). Advances in Robot Kinematics: Analysis and Control.

[15] Karger A., Röschel O., Vogler H. (1997). Proceedings of 107 Jahre Drehfluchtprinzip.

[16] Husty M., Karger A., Lenarcic J., Stanisic M.M. (2000). Advances in Robot Kinematics.

[17] Husty M., Karger A. (2001). CD-Rom Proceedings of the 10th International Workshop on Robotics–Adria–Danube Region RAAD'01, Vienna, Austria.

[18] Karger A. (2003). Architecture singular planar parallel manipulators. Mech. Mach. Theory.

[19] Karger A. (2008). Architecturally singular non-planar parallel manipulators. Mech. Mach. Theory.

[20] Nawratil G. (2008). On the degenerated cases of architecturally singular planar parallel manipulators. J. Geom. Graph..

[21] Mick S., Röschel O., Lenarcic J., Husty M.L. (1998). Advances in Robot Kinematics: Analysis and Control.

[22] Röschel O., Mick S., Lenarcic J., Husty M.L. (1998). Advances in Robot Kinematics: Analysis and Control.

[23] Nawratil G., Kecskemethy A., Müller A. (2009). Computational Kinematics.

[24] Wohlhart K. (2010). From higher degrees of shakiness to mobility. Mech. Mach. Theory.

[25] Karger A. (2001). Proceedings of Sborník 21. konference o geometrii a počítačové grafice.

[26] Bottema O., Roth B. (1979).

[27] Nawratil G. (2013). On elliptic self-motions of planar projective Stewart Gough platforms. Trans. Can. Soc. Mech. Eng..

[28] Nawratil G., Lenarcic J., Husty M. (2012). Latest Advances in Robot Kinematics.

[29] Kœnigs G. (1897).

[30] Borras J., Thomas F., Torras C. (2011). Architectural singularities of a class of pentapods. Mech. Mach. Theory.

[31] Nawratil G. (2012). Comments on “Architectural singularities of a class of pentapods”. Mech. Mach. Theory.

[32] Husty M.L. (1996). An algorithm for solving the direct kinematics of general Stewart–Gough platforms. Mech. Mach. Theory.

